# Short Versions of the Arabic Psychosocial Impact of Dental Aesthetics Questionnaire for Yemeni Adolescents: Cross-Sectional Derivation and Validation

**DOI:** 10.3390/children9030341

**Published:** 2022-03-02

**Authors:** Amal A. M. Alsanabani, Zamros Yuzadi Mohd Yusof, Wan Nurazreena Wan Hassan, Khalid Aldhorae, Helmi A. Alyamani

**Affiliations:** 1 Department of Community Oral Health and Clinical Prevention, Faculty of Dentistry, Universiti Malaya, Kuala Lumpur 50603, Malaysia; dr.amalali88@yahoo.com (A.A.M.A.); zamros@um.edu.my (Z.Y.M.Y.); 2 Department of Preventive Dentistry, Faculty of Dentistry, Sana’a University, Sana’a 2124, Yemen; 3 Department of Paediatric Dentistry and Orthodontics, Faculty of Dentistry, Universiti Malaya, Kuala Lumpur 50603, Malaysia; 4 Department of Orthodontics, College of Dentistry, University of Ibn al-Nafis for Medical Sciences, Sana’a 2124, Yemen; drdurai2008@gmail.com; 5 Dental Clinics, Basma Al Thuraya Medical Complex, Dammam 32273, Saudi Arabia; dr.helmialyamani@yahoo.com

**Keywords:** short version, oral health related quality of life, malocclusion, adolescent, validation

## Abstract

Objectives: To shorten the 24-item Arabic Psychosocial Impact of Dental Aesthetics Questionnaire (PIDAQ(A)) for adolescents in Yemen. Material and methods: Two shortening methods derived six-item and nine-item versions: the item impact method selected items with the highest impact scores as rated by 30 participants in each subscale; and the regression method was applied using data of 385 participants from the PIDAQ(A) validity study, with the total PIDAQ(A) score as the dependent variable, and its individual items as the independent variables. The four derived versions were assessed for validity and reliability. Results: The means of the six-item and nine-item short versions of both methods were close. Cronbach’s alpha values extended from 0.90 to 0.92 (intra-class correlations = 0.85–0.88). In criterion validity, strong significant correlations were detected between scores of all short versions and the 24-item PIDAQ(A) score (0.96–0.98; *p* < 0.001). Construct validity displayed significant associations among all short versions and self-perceived dental appearance rank and self-perceived need for orthodontic braces rank (*p* < 0.05). Mean scores of all short versions were significantly different between adolescents with severe malocclusion and those with slight malocclusion in discriminant validity tests. In conclusion, all PIDAQ(A) short versions are valid and reliable.

## 1. Introduction

Oral-health-related quality of life (OHRQoL) questionnaires are used to obtain patient-based outcomes that refer to the individual’s self-evaluation of the perception of a disease, and determine its perceived impact on quality of life [1]. In this context, OHRQoL measures tend to focus on the functional, psychological, and social impacts of the oral conditions on the life of the patient [2], and each of these instruments has its specific focus, purpose, and length [3].

Nevertheless, in epidemiological surveys, and even in clinical settings, the use of the questionnaire may be restricted by its length and the burden placed on persons [4]. Large scale population surveys are facing a persistent decrease in their response rates [5,6,7,8]. Various factors impact the decrease in response rates, including the effect of the instrument length [9]. Subsequently, item non-responses, if they occur, affect the validity of the results, and the utility of the data [10]. To address such situations, the application can be broadened by developing a short version of the questionnaire. This may lead to a reduction in the duration and financial costs of data collection. It can also lessen the risk of total and item non-responses [4]. Nevertheless, other studies mentioned that there is small or insignificant difference in the response rate between the short and long versions of a questionnaire [11,12,13]. Allen et al in 2020, [14] recommended that the long and short questionnaire versions should be included among survey design options. For clinical settings, using the short forms might be essential for practitioners where health care interventions should be targeted toward what the patients feel is important [15].

The Psychosocial Impact of Dental Aesthetics Questionnaire (PIDAQ) is a multidimensional instrument. It specifically aims to assess orthodontic aspects of OHRQoL [16]. PIDAQ was developed to be used among individuals seeking orthodontic treatment [17]. It consists of 23 items organized into four subscales: Dental Self-Confidence (six items); Social Impact (eight items); Psychological Impact (six items); and Aesthetic Concern (three items). PIDAQ version for adolescents aged 11 to 17 years old also showed good psychometric properties [18].

This questionnaire has been adapted in other languages for use by Malaysian [17,19], Spanish [20,21], Swedish [22], and Persian people [23]. It has also been cross-culturally adapted into an Arabic version, i.e. PIDAQ(A) to be filled by 12–17-year-old Yemeni children [24]. The PIDAQ(A) demonstrated good psychometric properties to be used for the Yemeni population with some modifications. The PIDAQ(A) consists of 24 items. These items are grouped into three subscales: Dental Self-Confidence (DSC) (6 items), Psychosocial Impact (PSI) (10 items), and Aesthetic Concern (AC) (8 items). The Cronbach’s α values for all subscales of the PIDAQ(A) were from 0.90 to 0.93 [24].

In Yemen, there is a need to have further information on the effects of malocclusion on the OHRQoL of the population regardless of the severity of the malocclusion. This may make important and relevant information for planning future dental services, as well as a projection of manpower needed to provide the service, more accessible. Therefore, it will provide information on the actual priority for orthodontic treatment needs. The importance of the PIDAQ instrument has led to the need to generate a short version of the PIDAQ(A) to be used in large scale surveys, and even in clinical settings. Another benefit of a short version of the PIDAQ(A) is that its use will minimize item non-response.

Therefore, the aim of this study was to derive the short versions of the PIDAQ(A) by using two item reduction methods. The objectives were to compare the content and psychometric properties of the short versions derived from the two methods, and to compare the short versions with the long version of the PIDAQ(A) [24]. The null hypothesis was that it would not be possible to shorten the questionnaire into valid and reliable short versions. The alternative hypothesis was that it would be possible to shorten the questionnaire into valid and reliable short versions.

## 2. Materials and Methods

### 2.1. Derivation of Short Version(s) of PIDAQ(A)

The PIDAQ(A) short versions were derived based on the processes used by a previous study [4]. The two methods were used with the intention of deriving a subset of 6- and 9-items that could capture as much information as possible from the 24-item PIDAQ(A).

#### 2.1.1. The Item Impact Method

The item impact method for generating short versions has used data acquired from the PIDAQ item impact study. 30 Yemeni school children, aged 12 to 17 years old, participated in a face-to-face interview. From the 30 participants, 56.7% were females and 43.3% were males. The participants were asked to specify which of the 24 items of the questionnaire describe problems that he/she had experienced in the past three months, and if yes, they were asked to rate the importance of the items on a four-point Likert scale ranging from a little bothered (score = 1), somewhat bothered (score = 2), bothered a lot (score = 3), to extremely bothered (score = 4) [4]. An impact score was calculated for each item by multiplying the item’s mean importance rating by the percentage of participants who reported positive responses [4]. Then, items were ranked within the three PIDAQ(A) subscales (DSC, PSI, and AC) according to their impact scores. The higher the impact score, the higher the level of psychosocial effect due to malocclusion problems. The final short version included the top 3- and 2-ranked items with the highest impact score in each subscale [4,25]. The 9-item and 6-item short versions utilizing the impact method are known as PIDAQ(A)-ISV9 and PIDAQ(A)-ISV6, respectively.

#### 2.1.2. The Regression Method

The regression method was applied to the data collected in a cross-sectional study of adolescents in Yemen to test the psychometric validations of the PIDAQ(A). Details of this study have been reported previously [24]. The short versions using stepwise regression were designated as the PIDAQ(A)-RSV9 and PIDAQ(A)-RSV6. The regression was used with the total PIDAQ(A) score obtained by adding the scores of all items as the dependent variable, and the independent variables were the individual items [4,26]. The first step to deriving the short version was generating a single model with all PIDAQ(A) items, and then a forward stepwise procedure with the aggregate for total score as the dependent variable was applied. The highest predictors of the overall score were recognized [4]. The stepwise regression was applied to result the adjusted R square (R^2^), and the estimated coefficients for all items. The top 3 and 2 items from each subscale that entered the model and produced the largest contribution to the coefficient of variation (R^2^) were selected for the PIDAQ(A)-RSV9 and PIDAQ(A)-RSV6, respectively.

### 2.2. The Psychometric Validations

The psychometric properties of the four PIDAQ(A) short versions comprised reliability, which involved the internal consistency and reproducibility, and testing the validity, which was achieved by criterion, convergent, and discriminant validities. The data used for this analysis were collected as part of the PIDAQ(A) 24-item questionnaire validation study in the cross-cultural adaptation work [24]. The PIDAQ(A) dataset included responses for 385 12–17-year-old Yemeni school adolescents. The proportion of females was higher (55.8%) than males (44.2%). The participants used the five-response Likert scale which has a rank order from 1 (never) to 5 (strongly agree) to rank their response to the long version (PIDAQ(A)-24), and each item results in a score from 1 to 5. All of their response scores for all items were combined into a final score. For the four short versions, scores were computed by summing up the scores of their items. The higher the total score, the higher the level of perception of being affected by malocclusion. Simultaneously, the clinical examination data for assessing the malocclusion for all of those participants were used in the analysis. In this clinical examination, the dental health and aesthetics components of the index of orthodontic treatment needs (IOTN-DHC and IOTN-AC), and the awareness component of the Perception of Occlusion Scale (POS), were measured [24].

A reliability assessment was conducted to examine the internal consistency and reproducibility for all short versions. Criterion validity was examined by determining the extent to which each of the short versions correlate with the PIDAQ(A)-24. In addition, criterion validity was estimated by testing the correlation between each short version score and scores of the Arabic version of Child Oral Impacts on Daily Performances (Child-OIDP) questionnaire [27]. The questionnaire of Child-OIDP assesses oral impact from malocclusion on eight daily activities, i.e., eating, speaking, cleaning teeth, relaxing, smiling, emotional stability, socializing, doing schoolwork, and socializing, where the “Position of the teeth” and “Spaces between” represent the malocclusion problems [28].

Convergent and discriminant validities, being the two dimensions of construct validity, were assessed by comparing the strength of associations of the four short versions with scores of global measures. For convergent validity, the associations of the short versions with perceived dental appearance were evaluated. Similarly, the associations between the short versions and satisfaction with dental appearance were also assessed. Participants ranked their response as excellent, good, average, or poor for perceived dental appearance, and very satisfied, satisfied, dissatisfied, or very dissatisfied for satisfaction with dental appearance [17,18,24].

For discriminant validity, the associations between the four short versions and each one of the following were assessed: self-rated perceived need (MI-S) and investigator-rated need for orthodontic treatment (MI-D), the dental health component of the index of orthodontic treatment needs (IOTN-DHC), and the perceived need for orthodontic treatment. In this study, the aesthetics component of the index of orthodontic treatment needs (IOTN-AC), and the awareness component of the Perception of Occlusion Scale (POS), represented the malocclusion index (MI-S and MI-D) [17,18,24]. Participants ranging in the upper quartile of the MI-S and MI-D were compared with their analogues ranging in the lower quartile. For perceived need for orthodontic treatment, the item response option was dichotomous (yes/no).

### 2.3. Statistical Analysis

IBM SPSS v.23 was used for data analysis. The analytic process performed in the study was identical to that used in our previous study [24] for assessing the association of short versions with other measures. For reliability analysis, the internal consistency was assessed for each of the PIDAQ(A) short versions by measuring the Cronbach’s α, Cronbach α if item deleted, inter-item correlation, and item-total correlation separately. For reproducibility, intraclass correlation coefficient (ICC) was calculated for the four PIDAQ(A) short versions. Bland and Altman analysis and paired *t*-test were performed to assess any significant change between the scores of the first and second administrations for each of the short versions [17,18,24].

For criterion validity, the correlation between the PIDAQ long version and each short version was determined using Spearman’s correlation, whereas the associations between each short version score and Child-OIDP total score was tested using the Pearson correlation coefficient. The score of Child-OIDP index was computed based on previous studies [17,18,24]. The frequency of the oral impact in any of the eight activities was multiplied by its severity. If there was no impact due to malocclusion, the score was recorded as 0. For convergent validity, the comparison of the PIDAQ short versions with the satisfaction with dental appearance and the perceived dental appearance were assessed by a Kruskal–Wallis test. For discriminant validity, comparisons of the relationships between scores of all short versions with the malocclusion index (MI-S and MI-D) were achieved using an independent *t*-test. The MI-S and MI-D malocclusion index, analysis of the severity of malocclusion, and effect size estimate were adapted from previous studies [17,18,24]. Cohen’s standardized effect size (ES) was computed to evaluate the difference between the measurements of the upper and lower groups [29]. ES could be considered as several levels of clinical meaningfulness (small: 0.2 ≤ ES < 0.5; moderate: 0.5 ≤ ES < 0.8; large: 0.8 ≤ ES) [29]. A comparison of the relationship between the short version scores with the IOTN-DHC was accomplished using the independent *t*-test, whereas the Mann–Whitney statistics were applied for the perceived need for orthodontic treatment. For the floor and ceiling effects, when more than 15% of participants had scores that were at the upper or lower limit, floor or ceiling effects were considered present, respectively [30].

Ethical approval was met for the primary study from the Faculty of Dentistry, Universiti Malaya, Malaysia, and the Faculty of Dentistry, Thamar University, Yemen.

## 3. Results

### 3.1. Derivation of the Short Versions PIDAQ(A)

Four short versions of the PIDAQ(A) were derived. Two versions (six and nine items) were developed using the item impact method, and the other two versions (six and nine items) were developed using the regression method [4].

#### 3.1.1. Item Impact Method

In the item impact method, the two or three items with the highest impact score from each of the three PIDAQ(A) subscales were included (Table 1). Item 23, item 6, and item 2 were the items with the highest impact scores: 1.817, 1.524, and 1.412 in the DSC, PSI, and AC subscales, respectively.

#### 3.1.2. Stepwise Regression Method

In the regression method, the top three and two items from each subscale (DSC, PSI, and AC) entering the model and making the largest contribution to the coefficient of variation (R2) were selected for the nine-item and six-item short versions, respectively. The order of the best predictors from PSI, DSC, and AC subscales were items 24, 14, and 6; items 16, 10, and 1; and items 21, 4, and 12, respectively (Table 2). In addition, Table 2 shows the coefficient (B) average for item predictors of the three PIDAQ(A) subscales. The regression coefficients (B) differ significantly from zero (*p* < 0.001), indicating a significant association between the predictors and the PIDAQ(A). All item predictors were positively related to the total PIDAQ(A) score.

#### 3.1.3. Content of the PIDAQ Short Versions

PIDAQ-ISV9 and PIDAQ-RSV9 are slightly similar where both of them shared five of their nine items, whereas the particular items for PIDAQ-ISV9 are 2, 1, 20, and 23, and the particular items for PIDAQ-RSV9 are 10, 14, 19, and 21 (Table 3, Supplemental Figures S1 and S3). PIDAQ-ISV6 and PIDAQ-RSV6 share four of their six items (Table 4, Supplemental Figures S2 and S4). PSI subscales of the six-item short versions were completely identical.

### 3.2. Descriptive Statistics

Table 5 shows that all PIDAQ(A) short versions revealed considerable variability in adolescents’ perceptions about the impact of malocclusion on their psychosocial condition. The means of the nine-item short versions were close to being similar, whereas their standard deviations (SD) were almost identical. Concerning the six-item short versions, their means and SD were almost identical. Floor and ceiling effects that represented the minimum and the maximum observed values were below the recommended maximum frequency of 15% in all short versions (Table 5).

PIDAQ(A)-ISV9 and PIDAQ(A)-RSV9 revealed that, respectively, 78.7% and 74.3% of the participants experienced malocclusion problems that impact “strongly and very strongly”. These proportions indicated that the PIDAQ(A)-ISV9 was more sensitive in detecting the most affected adolescents than PIDAQ(A)-RSV9. The PIDAQ(A)-ISV6 and PIDAQ(A)-RSV6 were slightly similar in their sensitivity in detecting the affected adolescents (60.5% and 61.0%). On the other hand, all short versions’ proportions were smaller than the PIDAQ(A) long version’s proportion, which was 85.5%.

### 3.3. Validity and Reliability

#### 3.3.1. Reliability

For the four short version questionnaires, the internal consistency, scale statistics, inter-item correlations, and Cronbach’s α (if item deleted) of the versions are provided in Table 6. The Cronbach’s alpha for the PIDAQ(A)-ISV9, PIDAQ(A)-RSV9, PIDAQ(A)-ISV6, and PIDAQ(A)-RSV6 were 0.92, 0.91, 0.90, and 0.90, respectively. They indicate all PIDAQ(A) short versions achieved high internal consistency reliability. None of the item total correlations scores were < 0.30 for all short versions. Likewise, for all short versions, none of the inter-item correlation scores were ≥0.90 or ≤0.30, except for PIDAQ(A)-RSV9, where its lowest value was 29 (between items 7 and 19).

Reproducibility test findings of the four short versions are shown in Table 7. The ICC values of all short versions were above 0.80 (*p* < 0.05). No statistically significant differences were detected between test and retest (T1 and T2) for the RSV-9 version only. The scores of the smallest detectable change (SDC) were lower in the regression short versions (RSV-9 and RSV-6). A Bland and Altman analysis showed that more than 90% of the scores of the second reproducibility test (T2) were within the limits of agreement for all versions.

#### 3.3.2. Criterion Validity

Table 8 shows the correlations between all short versions of PIDAQ(A) and the PIDAQ(A) long-version. The results showed that the correlations between the 4 short versions and the long-version instrument were almost perfect. The correlation coefficients for all versions were nearly identical to each other. The correlation coefficient for the PIDAQ(A)-RSV9 was the highest (rho: 0.983).

The association between ISV-9, RSV-9, ISV-6, and RSV-6 scores and Child-OIDP performance scores were statistically significant (*p* < 0.001). All PIDAQ(A) short versions showed a significant higher mean score for participants with Child-OIDP impact. The Pearson correlation coefficient showed that the Child-OIDP performance scores had moderate positive, statistically significant correlations with ISV-9, RSV-9, ISV-6, and RSV-6 mean scores. The correlation coefficient with the Child-OIDP performance score for ISV-9, ISV-6, RSV-9, and RSV-6 were slightly identical (Table 9).

#### 3.3.3. Convergent Validity

For convergent validity, the 4 short versions and self-perceived dental appearance were significantly associated (*p* < 0.01) (Table 10). In a similar vein, all short versions were significantly associated with perceived satisfaction with dental appearance (*p* < 0.01) (Table 11). The trends in self-perceived dental appearance and satisfaction were statistically significant.

#### 3.3.4. Discriminant Validity

The results of the discriminant validity showed that the mean scores of the short version questionnaires gradually increased with an increasing severity of malocclusion. Severity of malocclusion was illuminated by the investigator-rated (MI-D) and self-rated (MI-S) malocclusion scales. For both indices, statistically significant differences (*p* < 0.01) were shown between participants who were reported with no or slight malocclusion, and those reported with severe malocclusions (Table 12).

Table 13 presents the associations between the four short versions and the self-perceived need for braces. There was a significant association between self-perceived need for braces and the four short versions respectively. The results also showed that the associations between IOTN-DHC and PIDAQ(A) short versions were statistically significant (Table 14). There were statistically significant differences in the mean scores of the short versions between adolescents reported by the investigator having little grade of malocclusion and the adolescents with very great of malocclusions amongst the four short versions (*p* < 0.01).

As an ultimate corollary of the findings, for all PIDAQ(A) short versions, when the mean score increased, the effects of malocclusion problems on Yemeni adolescents were higher. In addition, the correspondence between the four short versions and the long version of PIDAQ(A) was high.

## 4. Discussion

The original PIDAQ instrument is a 23-item self-reporting questionnaire assessing the extent to which a person has experienced psychosocial problems due to malocclusion. The PIDAQA(A) was validated in the first part of our study [24]. In this study, the PIDAQ(A) short versions have been derived, tested for the psychometric validation, and compared with the 24-item PIDAQ(A).

This study produced four short versions of the PIDAQ(A). They were referred as PIDAQ(A)-ISV-9, PIDAQ(A)-RSV-9, PIDAQ(A)-ISV-6, and PIDAQ(A)-RSV-6. The methods used to produce the short versions followed those in the previous studies [4,15], and the item impact method and the regression method were applied. Concurrently, each of the shortening techniques that were used produced nine-item and a six-item measures. As a result, two or three items per each subscale were considered as the minimum number of items. The item impact method involved reduction of the items from the PIDAQ(A)-24 to include items that showed the highest impact scores in each subscale, whereas in the forward regression method, the first three and two items from each subscale entering the model, and which produced the highest adjusted R^2^, were selected. Two items per subscale were considered the minimum number of items [4].

As recommended by Jokovic et al. [4], the shortening of an instrument should take into account using more than one method to ascertain the effect of the approach on outcomes, because different methods can produce various short version measures, which may differ in items and properties. Therefore, this study had used two different methods to generate the short version.

Shortening a questionnaire is an effective way to increase the response rate [10,31]. Sahlqvist et al. [10] reported in their study that shortening a relatively lengthy instrument significantly increased the responses. Findings of their study revealed that an increase in the response rate was due to the shortening of the original questionnaire. Also, using the short version will be a useful substitute to the long questionnaire when time and financial costs are limited.

To date, there is no literature support for shortening the PIDAQ instrument, but there are other OHRQoL measures which have been shortened, such as the short versions of the Child Perceptions Questionnaire (CPQ11–14) [4,32] and the Oral Health Impact Profile (OHIP) Questionnaire, which underwent item reductions [33,34,35,36].

The results of the study showed that the PIDAQ(A) short versions exhibited considerable sensitivity, where proportions of PIDAQ(A)-ISV-9, PIDAQ(A)-RSV-9, PIDAQ(A)-ISV-6, and PIDAQ(A)-RSV-6 (78.7%, 74.3%, 61.5%, and 61.0%, respectively) revealed that the short versions of questionnaires detected substantial variability in adolescents’ perceptions of the effect of malocclusion in their life. In comparing their values, PIDAQ(A)-ISV9 was the highest (78.7%). Therefore, it seems reasonable to assert that the selected items for the short versions concern the most frequent and annoying problems reported by adolescents. However, values of all short versions were smaller than the long questionnaire (85.5%).

The psychometric properties have been achieved in terms of reliability (internal consistency and reproducibility) and validity (criterion, convergent, and discriminant validities). The short versions were comparable with the long version of the PIDAQ(A) in terms of sensitive and discriminant validities. This assessment is based on the responses from 385 children who were the participants of the main study.

For internal consistency, the ISV-9 and RSV-9 versions have almost similar Cronbach’s α values, (0.92) and (0.91), respectively, whereas the ISV-6 and RSV-6 versions showed identical Cronbach’s α values (0.90). The ICC-score-evaluated reproducibility was generally good for the four short versions, with scores ranging from 0.90 to 0.92 for all short versions. These scores were slightly similar to those in the PIDAQ(A) long version, where all of its subscales values were between 0.89 and 0.96 [24]. Nevertheless, RSV-9 version was better than others in the reproducibility test, and the RSV-9 version was the only version that showed no statistically significant differences between test and retest administrations. Assessment of criterion validity by comparing the correlation between all short versions and PIDAQ(A)-24 revealed that the correlation coefficient was highest for PIDAQ(A)-RSV9 (rho: 0.983). Whereas the correlation coefficient for the PIDAQ(A)-ISV9 was lesser (rho: 0.969). In addition, in criterion validity, the correlations between the short versions’ scores with the Child-OIDP score were shown to be statistically significant for all short versions (*p* < 0.001). The correlation coefficient with the Child-OIDP performance scores for the four short versions were slightly similar, but the ISV-9 was the highest (0.656). For construct validity, an analysis of convergent validity, as well as discriminant validity, showed statistically significant differences in associations with other global scales in all short versions.

Consequently, the high correlations between the PIDAQ(A)-24 and the short versions suggest that they are measuring the same construct [4]. All short versions were examined for cross-sectional validation, and all short forms showed good validity and reliability. Thus, the null hypotheses were rejected. In accordance with the results of the study, the short versions were almost identical. It may be worth pointing out that though the nine-item short versions presented interesting items, the results showed that the further shortening of the questionnaire to a six-item questionnaire was also valid, and as reliable as the nine-item version. In regard to the presence of differences between them, these differences were mostly negligible. However, the regression short versions were slightly stronger when compared to the impact short versions in reproducibility and criterion validity, that can be elucidated by the fact the items identified for the regression short versions were those that elucidate the most discrepancy in the total scores of the PIDAQ(A)-24 item.

The final consideration is if the regression method is better than the item impact method, or vice versa. Coste et al. [37] took the view that the expert-based method is more expedient. The utility of the item impact method is in selecting the items which are considered more meaningful for the individuals who will be answering the questionnaire. These individuals may be considered to be experienced with the impact of the discussed circumstances on their quality of life [4]. On the contrary, the short version, which was developed by statistical considerations represented by the regression method, performed reasonably well [4]. Nonetheless, Locker and Allen [15] considered that the approach of generating a short version measure is less important than its properties and content, this consideration was assisted by the findings of this study.

Eventually, PIDAQ(A) short versions can be used to distinguish Yemeni adolescents on the impact on their dental aesthetics when there is difficulty in using the PIDAQ(A) long version. The optional limitation of the study was that no short versions were administrated on their own. For further studies, we recommend measuring any changes in response rate when using both the long and short versions of the PIDAQ(A). Close attention should be paid to whether using the PIDAQ(A) short versions increases the response rate or not.

## 5. Conclusions

The PIDAQ(A) short versions were empirically shown to be valid, reliable, and appropriate for use in cross-sectional studies among Yemeni adolescents. The short versions of the PIDAQ(A) appeared to have had no negative impact in the validity and reliability when compared with the long version. The regression short versions are slightly more recommended for use than the impact short versions.

## Figures and Tables

**Table 1:** Highest scores of the 24-item Arabic PIDAQ (Impact items method) (n = 30).

Subscale	Item	Frequency	Importance	Impact
**DSC**				
23	Find own teeth nice *	0.76	2.391	1.817
12	Pleased to see own teeth in mirror *	0.80	2.041	1.632
7	Like to show their teeth *	0.73	2.136	1.559
17	Teeth look nice to others	0.63	1.736	1.093
4	Proud of own teeth	0.50	1.533	0.766
**PSI**				
6	Distressed because of others’ nice teeth *	0.53	2.875	1.524
24	Not attractive because of own teeth *	0.43	3.077	1.323
20	Wish to look better *	0.36	2.909	1.047
22	Boys/girls find own teeth ugly	0.40	2.583	1.033
13	People look strange at my teeth	0.33	2.600	0.858
9	Teasing	0.23	3.285	0.755
14	Shy because of own teeth	0.26	2.875	0.748
11	Others have nicer teeth	0.26	2.750	0.715
5	What others think	0.30	2.333	0.699
3	Envy others for their teeth	0.20	3.167	0.633
**AC**				
2	Hold back their smile *	0.46	3.071	1.412
16	Feel bad about own teeth *	0.36	3.545	1.276
1	Do not like own teeth in mirror *	0.40	2.917	1.233
8	Do not like own teeth on photos	0.46	2.214	0.91
19	Stupid comments from others	0.26	3.090	0.803
10	Unhappy about own teeth	0.26	3.000	0.78
18	Do not like own teeth on video	0.23	1.56	0.36
15	Hiding own teeth	0.16	1.86	0.30

DSC: dental self-confidence; PSI: psychosocial impact; AC: aesthetic concern; * the selected items for the construct of the item impact short versions.

**Table 2:** Forward regression model: the total aggregate score for the PIDAQ(A) is the dependent variable, and all items are the independent variables (N = 385).

PIDAQ(A) Items		Unstandardized Coefficients B	t	*p* Value	95.0% Confidence Interval for B	
Subscale	Item				Lower Bound	Upper Bound
DSC						
21	Satisfied with own teeth’s appearance *	1.04	48.65	0.000 **	0.99	1.08
12	Pleased to see own teeth in mirror *	1.04	49.75	0.000 **	0.99	1.08
7	Like to show their teeth *	0.95	52.12	0.000 **	0.93	1.00
17	Teeth look nice to others	0.96	50.50	0.000 **	0.92	1.00
4	Proud of own teeth	0.99	53.80	0.000 **	0.96	1.03
23	Find own teeth nice	1.00	41.93	0.000 **	0.95	1.05
PSI						
24	Not attractive because of own teeth *	1.04	50.52	0.000 **	1.00	1.08
6	Distressed because of others’ nice teeth *	0.97	40.78	0.000 **	0.93	1.02
14	Shy because of own teeth *	1.02	37.87	0.000 **	0.97	1.07
9	Teasing	1.06	51.87	0.000 **	1.02	1.10
20	Wish to look better	0.90	62.38	0.000 **	0.96	1.02
22	Boys/girls find own teeth ugly	1.07	47.12	0.000 **	1.03	1.11
5	What others think	1.02	53.93	0.000 **	0.98	1.05
3	Envy others for their teeth	0.97	53.45	0.000 **	0.94	1.00
11	Others have nicer teeth	.98	63.29	0.000 **	0.96	1.02
13	People look strange at my teeth	1.03	40.20	0.000 **	0.98	1.01
AC						
16	Feel bad about own teeth *	0.95	33.86	0.000 **	1.00	1.01
10	Unhappy about own teeth *	0.96	37.72	0.000 **	0.91	1.01
19	Stupid comments from others *	0.96	65.44	0.000 **	0.94	0.99
1	Do not like own teeth in mirror	0.99	42.00	0.000 **	0.95	1.04
8	Do not like own teeth on photos	0.97	46.71	0.000 **	0.92	1.01
2	Hold back their smile	1.01	47.10	0.000 **	0.97	1.05
18	Do not like own teeth on video	1.04	42.83	0.000 **	0.99	1.09
15	Hiding own teeth	0.99	46.29	0.000 **	0.95	1.04

DSC: dental self-confidence; PSI: psychosocial impact; AC: aesthetic concern; * selected items for the regression short versions; ** *p* value < 0.001.

**Table 3:** Items of PIDAQ(A)-ISV9 and PIDAQ(A)-RSV9.

Subscale	ISV Particular Items	Common Items	RSV Particular Items
DSC			
	23. Find own teeth nice.	12. Pleased to see own teeth in mirror 7.Like to show their teeth.	21. Satisfied with own teeth’s appearance.
PSI			
	20. Wish to look better.	24. Not attractive because of own teeth.	14. Shy because of own teeth.
		6. Distressed because of others’ nice teeth.	
AC			
	2. Hold back their smile.	16. Feel bad about own teeth.	10. Unhappy about own teeth.
	1. Do not like own teeth in mirror.		19. Stupid comments from others

ISV9: nine-item impact short version; RSV9: nine-item regression short version; DSC: dental self-confidence; PSI: psychosocial impact; AC: aesthetic concern.

**Table 4:** Items of PIDAQ(A)-ISV6 and PIDAQ(A)-RSV6.

Subscale	ISV Particular Items	Common Items	RSV Particular Items
DSC			
	23. Find own teeth nice.	12. Pleased to see own teeth in mirror	21. Satisfied with own teeth’s appearance.
PSI			
		24. Not attractive because of own teeth.	
		6. Distressed because of others’ nice teeth.	
AC			
	2. Hold back their smile.	16. Feel bad about own teeth.	10. Unhappy about own teeth.

ISV6: six-item impact short version; RSV6: six-item regression short version; DSC: dental self-confidence; PSI: psychosocial impact; AC: aesthetic concern.

**Table 5:** Descriptive statistics for the PIDAQ(A)-ISV9, PIDAQ(A)-ISV6, PIDAQ(A)-RSV9, and PIDAQ(A)-RSV6.

Short Version	Mean (SD)	Range of Possible Scores	Range of Scores	% Floor	% Ceiling	Quartiles		
						Q1	Q2	Q3
PIDAQ(A)-ISV9	24.25 (8.86)	9–45	9–45	1.6	0.8	17.00	24.00	30.00
PIDAQ(A)-RSV9	22.30 (8.71)	9–45	9–45	2.6	0.5	15.00	21.00	27.00
PIDAQ(A)-ISV6	15.00 (6.05)	6–30	6–30	4.9	0.8	10.00	15.00	19.00
PIDAQ(A)-RSV6	14.68 (6.21)	6–30	6–30	6.5	0.8	9.00	14.00	19.00

SD: standard deviation; ISV9: nine-item impact short version; RSV9: nine-item regression short version; ISV6: six-item impact short version; RSV6: six-item regression short version; Q1: lower quartile; Q2: middle quartile; Q3: upper quartile.

**Table 6:** The internal consistency, scale statistics, and inter-item correlations of all short versions of the Arabic PIDAQ (n = 385).

Short Version	Cronbach’s α 385 (12–17 Years)	ICC (95% CI)	Inter-Item Correlations			Corrected Item Total Correlation		Cronbach’s α if Item Deleted
			Mean	Min	Max	Min	Max	
PIDAQ(A)-ISV9	0.92	0.88 (0.82–0.91)	2.69	0.39	0.73	0.59	0.81	0.90–0.91
PIDAQ(A)-RSV9	0.91	0.88 (0.83–0.91)	2.48	0.29	0.78	0.50	0.83	0.89–0.91
PIDAQ(A)-ISV6	0.90	0.85 (0.78–0.90)	2.50	0.48	0.76	0.65	0.81	0.86–0.89
PIDAQ(A)-RSV6	0.90	0.86 (0.79–0.91)	2.45	0.51	0.76	0.67	0.81	0.88–0.90

ICC: intraclass correlation coefficient; 95% IC: 95% confidence interval; ISV9: nine-item impact short version; RSV9: nine-item regression short version; ISV6: six-item impact short version; RSV6: six-item regression short version.

**Table 7:** The reproducibility assessment for the Arabic PIDAQ short versions.

Short Version	ICC Agreement (95% CI)	SEM	SDC	Paired *t*-Test		Bland and Altman		
				MDiff	(SD)	95% Limits of Agreement		
						Lower	Upper	%Within Limits
PIDAQ(A)-ISV9	0.92 (0.88–0.94)	0.65	1.80	−0.91 *	4.22	−9.18	7.36	96.6
PIDAQ(A)-RSV9	0.92 (0.89–0.95)	0.52	1.44	−0.73	4.19	−8.94	7.48	90.7
PIDAQ(A)-ISV6	0.90 (0.86–0.93)	0.59	1.63	−0.83 *	3.04	−6.79	5.13	94.0
PIDAQ(A)-RSV6	0.91 (0.86–0.94)	0.43	1.19	−0.60 *	2.92	−6.32	5.12	95.7

* *p* < 0.05 (paired-sample *t*-test); CI: confidence interval; SEM: standard error of measurement; SDC: smallest detectable change; MDiff: mean differences; SD: standard deviation; ISV9: nine-item impact short version; RSV9: nine-item regression short version; ISV6: six-item impact short version; RSV6: six-item regression short version.

**Table 8:** The criterion validity: correlations between the short versions and the long version of PIDAQ(A) (n = 385).

Short-Form	Long-Form PIDAQ(A)	*p* Value
	rho	
PIDAQ(A)-ISV9	0.969	0.000 **
PIDAQ(A)-RSV9	0.983	0.000 **
PIDAQ(A)-ISV6	0.960	0.000 **
PIDAQ(A)-RSV6	0.969	0.000 **

rho: Spearman’s correlation coefficient; ** *p* value < 0.001 for all short versions; ISV9: nine-item impact short version; RSV9: nine-item regression short version; ISV6: six-item impact short version; RSV6: six-item regression short version.

**Table 9:** The criterion validity: correlations of all PIDAQ(A) short versions and Child-OIDP questionnaire (n = 385).

PIDAQ(A) Short Version	Child-OIDP Prevalence	N	Mann–Whitney U		Quartiles			Pearson Correlation		
			SV Score					*p* Value	Child-OIDP Performance	*p* Value
			Mean	SD	Q1	Q2	Q3			
**ISV9**	No	200	17.86	5.12	14.00	18.00	22.00	0.000 **	0.656	0.000 **
	Yes	185	31.16	6.57	26.00	31.00	37.00			
**RSV9**	No	200	15.52	5.01	11.00	15.00	19.00	0.000 **	0.635	0.000 **
	Yes	185	29.04	7.37	24.00	28.00	35.00			
**ISV6**	No	200	10.73	3.33	8.00	10.00	13.00	0.000 **	0.642	0.000 **
	Yes	185	19.62	4.80	16.00	19.00	23.00			
**RSV6**	No	200	9.77	3.48	7.00	9.00	12.00	0.000 **	0.625	0.000 **
	Yes	185	19.18	5.23	15.00	18.00	23.00			

SD: standard deviation; scores of the DSC subscale items were reversed; SV score: short version score; ISV9: nine-item impact short version; RSV9: nine-item regression short version; ISV6: six-item impact short version; RSV6: six-item regression short version; Q1: lower quartile; Q2: middle quartile; Q3: upper quartile; ** *p* value < 0.001 for all short versions.

**Table 10:** The convergent validity: association between all PIDAQ(A) short versions with self-perceived dental appearance rank (n = 385).

Short Version	Appearance Rating	N	Scores of Questionnaire		Quartiles			*p* Value
			Mean	SD	Q1	Q2	Q3	
PIDAQ-ISV-9	Excellent	100	15.07	3.90	12.00	15.00	18.00	0.000 **
	Good	115	21.50	4.88	18.00	22.00	25.00	
	Average	109	28.09	5.56	25.00	29.00	31.00	
	Poor	61	37.59	3.76	35.00	38.00	40.00	
PIDAQ-RSV-9	Excellent	100	12.79	3.39	10.00	12.00	15.00	0.000 **
	Good	115	18.79	4.96	15.00	19.00	23.00	
	Average	109	25.78	5.78	22.00	26.00	29.00	
	Poor	61	36.38	4.41	34.00	37.00	39.50	
PIDAQ-ISV-6	Excellent	100	8.91	2.38	7.00	9.00	10.00	0.000 **
	Good	115	13.03	3.27	10.00	13.00	15.00	
	Average	109	17.50	3.97	15.00	17.00	20.00	
	Poor	61	24.25	2.92	22.00	24.00	27.00	
PIDAQ-RSV-6	Excellent	100	7.95	2.22	6.00	7.00	9.00	0.000 **
	Good	115	11.93	3.48	9.00	12.00	15.00	
	Average	109	16.97	4.17	14.00	17.00	19.00	
	Poor	61	24.34	3.20	22.00	25.00	27.00	

Sores of the DSC subscale items were reversed; SD: standard deviation; ISV9: nine-item impact short version; RSV9: nine-item regression short version; ISV6: six-item impact short version; RSV6: six-item regression short version; Q1: lower quartile; Q2: middle quartile; Q3: upper quartile; ** *p* value < 0.001 for all PIDAQ(A) short versions.

**Table 11:** The convergent validity: association between all short versions of Arabic PIDAQ with perceived satisfaction with dental appearance rank (n = 385).

Short Version	Satisfaction Rating	N	Scores of Questionnaire		Quartiles			*p* Value
			Mean	SD	Q1	Q2	Q3	
PIDAQ-ISV-9	Very satisfied	91	15.07	3.95	12.00	15.00	18.00	0.000 **
	Satisfied	115	20.49	4.70	17.00	21.00	24.00	
	Dissatisfied	119	28.24	5.08	25.00	29.00	31.00	
	Very dissatisfied	60	37.47	5.07	36.00	38.00	40.75	
PIDAQ-RSV-9	Very satisfied	91	12.65	3.09	10.00	12.00	14.00	0.000 **
	Satisfied	155	17.97	4.87	15.00	18.00	22.00	
	Dissatisfied	119	25.91	5.49	22.00	26.00	29.00	
	Very dissatisfied	60	36.23	5.31	34.00	37.00	39.00	
PIDAQ-ISV-6	Very satisfied	91	8.88	2.37	7.00	9.00	10.00	0.000 **
	Satisfied	115	12.47	3.18	10.00	12.00	15.00	
	Dissatisfied	119	24.23	3.67	22.00	25.00	27.00	
	Very dissatisfied	60	24.23	3.67	22.00	25.00	27.00	
PIDAQ-RSV-6	Very satisfied	91	7.81	1.91	6.00	7.00	9.00	0.000 **
	Satisfied	115	11.36	3.36	9.00	12.00	14.00	
	Dissatisfied	119	17.04	4.05	14.00	17.00	19.00	
	Very dissatisfied	60	24.28	3.50	23.00	25.00	27.00	

Scores of the DSC subscale items were reversed; SD: standard deviation; ISV9: nine-item impact short version; RSV9: nine-item regression short version; ISV6: six-item impact short version; RSV6: six-item regression short version; Q1: lower quartile; Q2: middle quartile; Q3: upper quartile; ** *p* value < 0.001 for all PIDAQ(A) short versions.

**Table 12:** The discriminant validity: all short versions in association with self-rated (MI-S) and interviewer-rated (MI-D) malocclusion (n = 385).

(MI-S)	Quartile		ES	*p* Value
	Q1	Q3		
No.	122	129		
Short Version	Mean (SD)	Mean (SD)		
PIDAQ(A)-ISV9	16.43 (4.57)	33.70 (5.98)	−3.25	0.000 **
PIDAQ(A)-RSV9	14.24 (4.30)	31.74 (6.81)	−3.07	0.000 **
PIDAQ(A)-ISV6	9.88 (2.88)	21.40 (4.47)	−3.06	0.000 **
PIDAQ(A)-RSV6	8.78 (2.92)	21.03 (4.87)	−3.05	0.000 **
**(MI-D)**	**Quartile**		**ES**	***p* Value**
	**Q1**	**Q3**		
**No.**	**121**	**130**		
**Short Version**	**Mean (SD)**	**Mean (SD)**		
PIDAQ(A)-ISV9	19.21(6.76)	27.28 (8.64)	−1.04	0.000 **
PIDAQ(A)-RSV9	16.96 (6.80)	24.86 (9.07)	−0.99	0.000 **
PIDAQ(A)-ISV6	11.61(4.41)	17.08 (6.01)	−1.04	0.000 **
PIDAQ(A)-RSV6	10.79 (4.85)	16.25 (6.31)	−0.97	0.000 **

(MI-S) self-rated index; (MI-D) investigator-rated index; ** *p* value < 0.001 for all short versions; ISV9: nine-item impact short version; RSV9: nine-item regression short version; ISV6: six-item impact short version; RSV6: six-item regression short version; ES: effect size; Q1: lower quartile; Q3: upper quartile.

**Table 13:** The discriminant validity: associations of all Arabic PIDAQ short versions with regards to ranking the need for braces (n = 385).

Short Versions	Need Braces	N	SV Scores		Quartiles			*p* Value
			Mean	SD	Q1	Q2	Q3	
**PIDAQ(A)-ISV9**	Yes	199	30.20	7.07	25.00	29.00	36.00	0.000 **
	No	186	17.88	5.53	13.75	18.00	21.00	
**PIDAQ(A)-RSV9**	Yes	199	27.93	7.89	23.00	27.00	35.00	0.000 **
	No	186	15.69	5.63	11.00	15.00	19.00	
**PIDAQ(A)-ISV6**	Yes	199	18.93	5.12	15.00	18.00	23.00	0.000 **
	No	186	10.80	3.66	8.00	9.00	13.00	
**PIDAQ(A)-RSV6**	Yes	199	18.44	5.57	14.00	18.00	23.00	0.000 **
	No	186	9.85	3.86	7.00	9.00	12.00	

** *p* value < 0.001 for all PIDAQ(A) short versions; SV score: short version score; ISV9: nine-item impact short version; RSV9: nine-item regression short version; ISV6: six-item impact short version; RSV6: six-item regression short version; Q1: lower quartile; Q2: middle quartile; Q3: upper quartile.

**Table 14:** The discriminant validity: associations of the short versions with IOTN-DHC.

Short Version	Occlusal Traits	N	SV Scores		Quartiles			*p* Value
			Mean	SD	Q1	Q2	Q3	
PIDAQ(A)-ISV9	No/Little	65	16.02	4.79	12.00	16.00	19.00	0.000 **
	Moderate	186	20.98	6.01	16.00	21.00	25.00	
	Great	120	32.20	6.59	27.25	32.00	37.00	
	Very great	14	37.71	4.95	35.25	38.50	41.25	
PIDAQ(A)-RSV9	No/Little	65	13.55	4.11	10.50	13.00	15.50	0.000 **
	Moderate	186	18.61	6.04	13.75	18.00	23.25	
	Great	120	30.25	7.48	25.00	31.00	36.00	
	Very great	14	36.00	5.75	30.75	36.50	41.25	
PIDAQ(A)-ISV6	No/Little	65	9.40	2.83	7.00	9.00	11.00	0.000 **
	Moderate	186	12.76	3.96	9.00	13.00	16.00	
	Great	120	20.47	4.79	17.00	21.00	24.00	
	Very great	14	24.00	3.86	21.00	25.00	26.00	
PIDAQ(A)-RSV6	No/Little	65	8.40	2.66	6.00	8.00	9.00	0.000 **
	Moderate	186	11.89	4.23	8.00	12.00	15.00	
	Great	120	20.13	5.32	16.00	21.00	24.00	
	Very great	14	23.57	4.07	20.00	24.50	27.25	

IOTN-DHC: Dental Health Component of Index of Orthodontic Treatment Need; SV score: short version score; ISV9: nine-item impact short version; RSV9: nine-item regression short version; ISV6: six-item impact short version; RSV6: six-item regression short version; Q1: lower quartile; Q2: middle quartile; Q3: upper quartile; ** *p* value < 0.001 for all PIDAQ(A) short versions.

## Data Availability

Not applicable.

## Supplementary Materials

The following supporting information can be downloaded at: https://www.mdpi.com/article/10.3390/children9030341/s1, Figure S1: Item Impact Short Version-9 item; Figure S2: Regression Short Version-9 item, Figure S3: Item Impact Short Version-6 item, Figure S4: Regression Short Version-6 item.
