# Supplementary figures and images for: Short Versions of the Arabic Psychosocial Impact of Dental Aesthetics Questionnaire for Yemeni Adolescents: Cross-Sectional Derivation and Validation
Source: Children (Basel). 2022 Mar 2;9(3):341. doi: 10.3390/children9030341 (PMC8947303; doi:10.3390/children9030341)

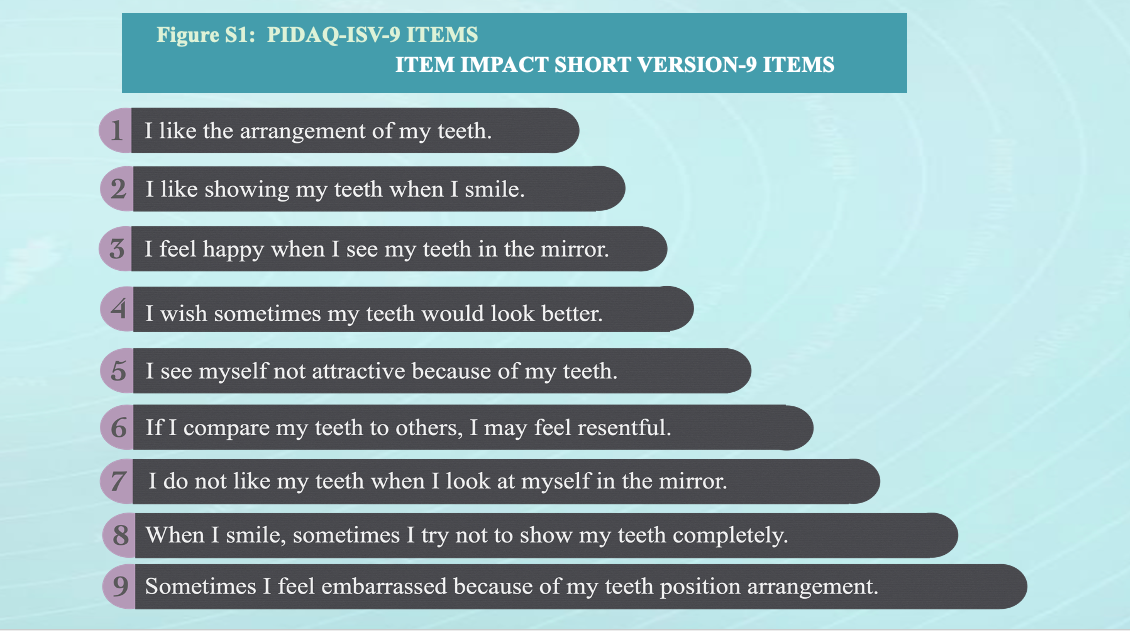

Supplement: Supplementary file 1 [file children-09-00341-s001.zip › supplementary/Figure S1. ITEM IMPACT SHORT VERSION-9 ITEMS.png]

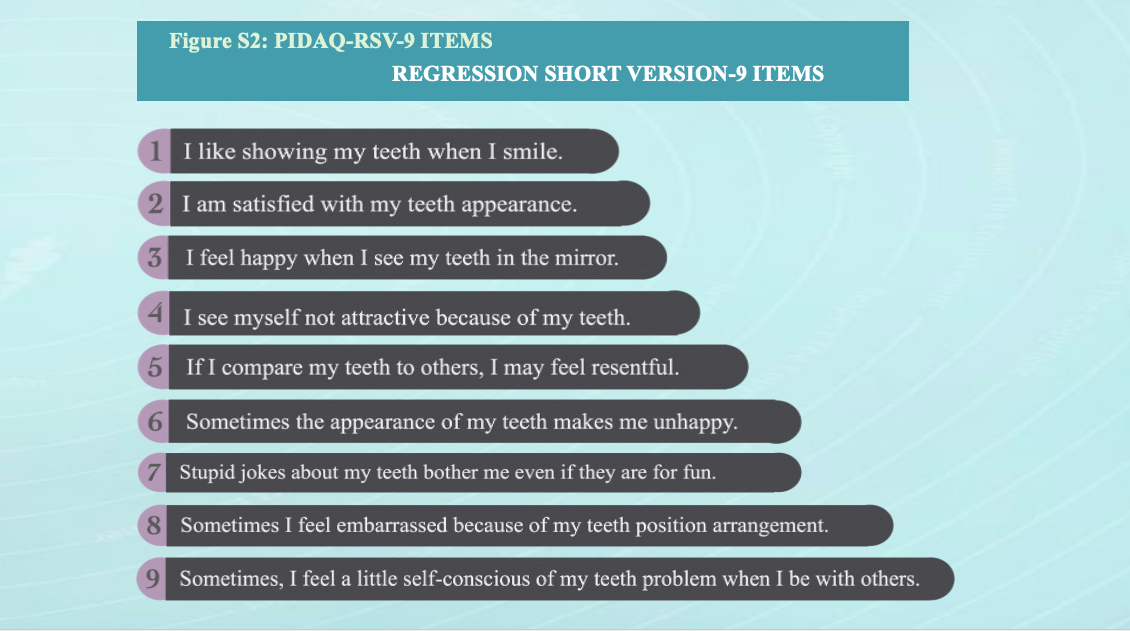

Supplement: Supplementary file 1 [file children-09-00341-s001.zip › supplementary/Figure S2. REGRESSION SHORT VERSION-9 ITEMS.png]

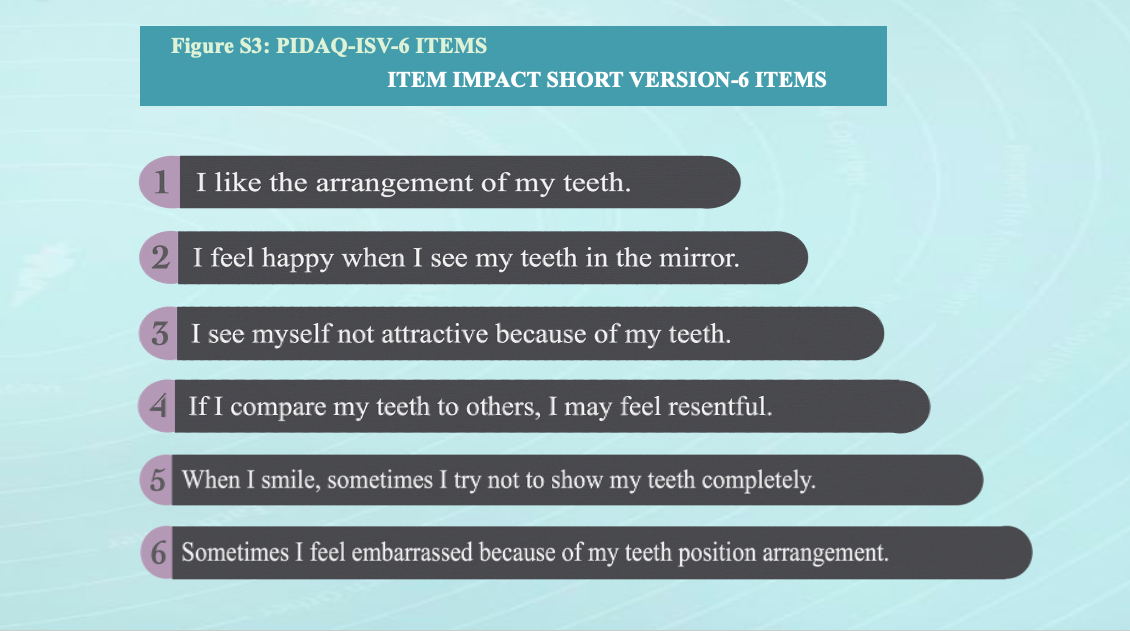

Supplement: Supplementary file 1 [file children-09-00341-s001.zip › supplementary/Figure S3. ITEM IMPACT SHORT VERSION-6 ITEMS.png]

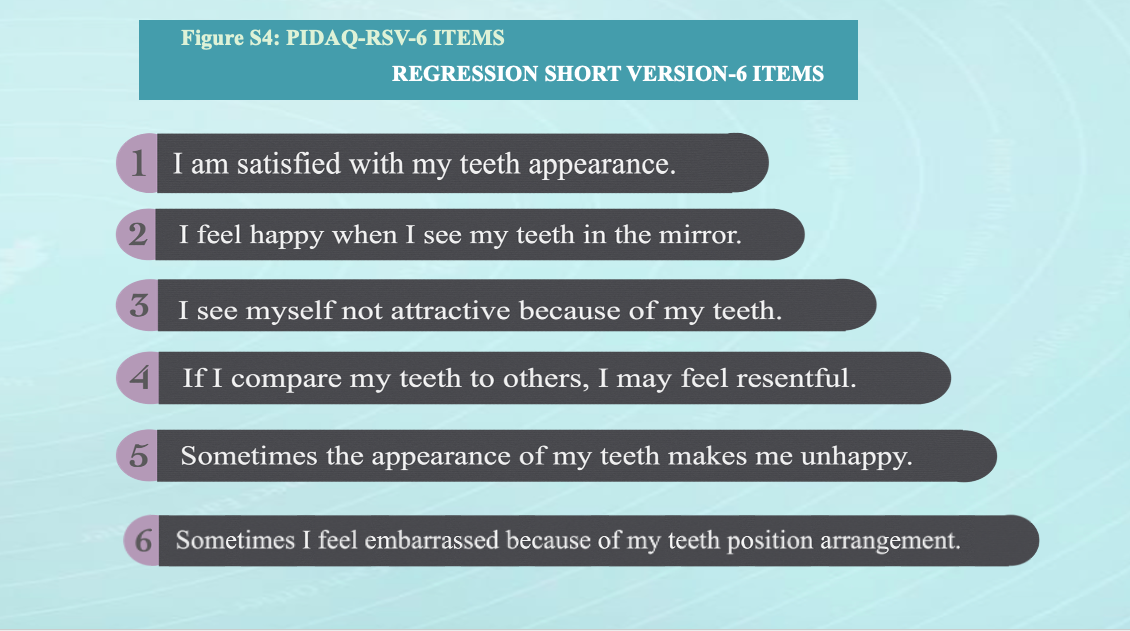

Supplement: Supplementary file 1 [file children-09-00341-s001.zip › supplementary/Figure S4. REGRESSION SHORT VERSION-6 ITEMS.png]
